# Supplementary material for: An open-source framework for large-scale, flexible evaluation of biomedical text mining systems
Source: J Biomed Discov Collab. 2008 Jan 29;3:1. doi: 10.1186/1747-5333-3-1 (PMC2276192; doi:10.1186/1747-5333-3-1)
Supplement: Additional file 1 — Evaluation platform source code. The additional file contains the software evaluation framework discussed in this paper. The most current release can be downloaded from SourceForge.net [1]. See the accompanying README file for instructions on installation and use. Also included in the distribution is a collection of UIMA wrappers for some commonly used BioNLP tools and annotated corpora. [file 1747-5333-3-1-S1.zip › bionlp_uima_repository-1.0.1/docs/api/src-html/edu/uchsc/ccp/uima/annotation/syntactic/CCPTokenAnnotation_Type.html]

```
001    
002    /* First created by JCasGen Tue Aug 28 16:44:10 MDT 2007 */
003    package edu.uchsc.ccp.uima.annotation.syntactic;
004    
005    import org.apache.uima.cas.Feature;
006    import org.apache.uima.cas.FeatureStructure;
007    import org.apache.uima.cas.Type;
008    import org.apache.uima.cas.impl.CASImpl;
009    import org.apache.uima.cas.impl.FSGenerator;
010    import org.apache.uima.cas.impl.FeatureImpl;
011    import org.apache.uima.cas.impl.TypeImpl;
012    import org.apache.uima.jcas.JCas;
013    import org.apache.uima.jcas.JCasRegistry;
014    
015    import edu.uchsc.ccp.uima.annotation.CCPSyntacticAnnotation_Type;
016    
017    /** This annotation type is used to mark up tokens.
018     * Updated by JCasGen Tue Aug 28 16:44:10 MDT 2007
019     * @generated */
020    public class CCPTokenAnnotation_Type extends CCPSyntacticAnnotation_Type {
021      /** @generated */
022      protected FSGenerator getFSGenerator() {return fsGenerator;}
023      /** @generated */
024      private final FSGenerator fsGenerator = 
025        new FSGenerator() {
026          public FeatureStructure createFS(int addr, CASImpl cas) {
027                             if (CCPTokenAnnotation_Type.this.useExistingInstance) {
028                               // Return eq fs instance if already created
029                         FeatureStructure fs = CCPTokenAnnotation_Type.this.jcas.getJfsFromCaddr(addr);
030                         if (null == fs) {
031                           fs = new CCPTokenAnnotation(addr, CCPTokenAnnotation_Type.this);
032                               CCPTokenAnnotation_Type.this.jcas.putJfsFromCaddr(addr, fs);
033                               return fs;
034                         }
035                         return fs;
036            } else return new CCPTokenAnnotation(addr, CCPTokenAnnotation_Type.this);
037              }
038        };
039      /** @generated */
040      public final static int typeIndexID = CCPTokenAnnotation.typeIndexID;
041      /** @generated 
042         @modifiable */
043      public final static boolean featOkTst = JCasRegistry.getFeatOkTst("edu.uchsc.ccp.uima.annotation.syntactic.CCPTokenAnnotation");
044     
045      /** @generated */
046      final Feature casFeat_partOfSpeech;
047      /** @generated */
048      final int     casFeatCode_partOfSpeech;
049      /** @generated */ 
050      public String getPartOfSpeech(int addr) {
051            if (featOkTst && casFeat_partOfSpeech == null)
052          jcas.throwFeatMissing("partOfSpeech", "edu.uchsc.ccp.uima.annotation.syntactic.CCPTokenAnnotation");
053        return ll_cas.ll_getStringValue(addr, casFeatCode_partOfSpeech);
054      }
055      /** @generated */    
056      public void setPartOfSpeech(int addr, String v) {
057            if (featOkTst && casFeat_partOfSpeech == null)
058          jcas.throwFeatMissing("partOfSpeech", "edu.uchsc.ccp.uima.annotation.syntactic.CCPTokenAnnotation");
059        ll_cas.ll_setStringValue(addr, casFeatCode_partOfSpeech, v);}
060        
061      
062     
063      /** @generated */
064      final Feature casFeat_stem;
065      /** @generated */
066      final int     casFeatCode_stem;
067      /** @generated */ 
068      public String getStem(int addr) {
069            if (featOkTst && casFeat_stem == null)
070          jcas.throwFeatMissing("stem", "edu.uchsc.ccp.uima.annotation.syntactic.CCPTokenAnnotation");
071        return ll_cas.ll_getStringValue(addr, casFeatCode_stem);
072      }
073      /** @generated */    
074      public void setStem(int addr, String v) {
075            if (featOkTst && casFeat_stem == null)
076          jcas.throwFeatMissing("stem", "edu.uchsc.ccp.uima.annotation.syntactic.CCPTokenAnnotation");
077        ll_cas.ll_setStringValue(addr, casFeatCode_stem, v);}
078        
079      
080     
081      /** @generated */
082      final Feature casFeat_lemma;
083      /** @generated */
084      final int     casFeatCode_lemma;
085      /** @generated */ 
086      public String getLemma(int addr) {
087            if (featOkTst && casFeat_lemma == null)
088          jcas.throwFeatMissing("lemma", "edu.uchsc.ccp.uima.annotation.syntactic.CCPTokenAnnotation");
089        return ll_cas.ll_getStringValue(addr, casFeatCode_lemma);
090      }
091      /** @generated */    
092      public void setLemma(int addr, String v) {
093            if (featOkTst && casFeat_lemma == null)
094          jcas.throwFeatMissing("lemma", "edu.uchsc.ccp.uima.annotation.syntactic.CCPTokenAnnotation");
095        ll_cas.ll_setStringValue(addr, casFeatCode_lemma, v);}
096        
097      
098     
099      /** @generated */
100      final Feature casFeat_tokenNumber;
101      /** @generated */
102      final int     casFeatCode_tokenNumber;
103      /** @generated */ 
104      public int getTokenNumber(int addr) {
105            if (featOkTst && casFeat_tokenNumber == null)
106          jcas.throwFeatMissing("tokenNumber", "edu.uchsc.ccp.uima.annotation.syntactic.CCPTokenAnnotation");
107        return ll_cas.ll_getIntValue(addr, casFeatCode_tokenNumber);
108      }
109      /** @generated */    
110      public void setTokenNumber(int addr, int v) {
111            if (featOkTst && casFeat_tokenNumber == null)
112          jcas.throwFeatMissing("tokenNumber", "edu.uchsc.ccp.uima.annotation.syntactic.CCPTokenAnnotation");
113        ll_cas.ll_setIntValue(addr, casFeatCode_tokenNumber, v);}
114        
115      
116     
117      /** @generated */
118      final Feature casFeat_typedDependencies;
119      /** @generated */
120      final int     casFeatCode_typedDependencies;
121      /** @generated */ 
122      public int getTypedDependencies(int addr) {
123            if (featOkTst && casFeat_typedDependencies == null)
124          jcas.throwFeatMissing("typedDependencies", "edu.uchsc.ccp.uima.annotation.syntactic.CCPTokenAnnotation");
125        return ll_cas.ll_getRefValue(addr, casFeatCode_typedDependencies);
126      }
127      /** @generated */    
128      public void setTypedDependencies(int addr, int v) {
129            if (featOkTst && casFeat_typedDependencies == null)
130          jcas.throwFeatMissing("typedDependencies", "edu.uchsc.ccp.uima.annotation.syntactic.CCPTokenAnnotation");
131        ll_cas.ll_setRefValue(addr, casFeatCode_typedDependencies, v);}
132        
133       /** @generated */
134      public int getTypedDependencies(int addr, int i) {
135            if (featOkTst && casFeat_typedDependencies == null)
136          jcas.throwFeatMissing("typedDependencies", "edu.uchsc.ccp.uima.annotation.syntactic.CCPTokenAnnotation");
137        if (lowLevelTypeChecks)
138          return ll_cas.ll_getRefArrayValue(ll_cas.ll_getRefValue(addr, casFeatCode_typedDependencies), i, true);
139        jcas.checkArrayBounds(ll_cas.ll_getRefValue(addr, casFeatCode_typedDependencies), i);
140            return ll_cas.ll_getRefArrayValue(ll_cas.ll_getRefValue(addr, casFeatCode_typedDependencies), i);
141      }
142       
143      /** @generated */ 
144      public void setTypedDependencies(int addr, int i, int v) {
145            if (featOkTst && casFeat_typedDependencies == null)
146          jcas.throwFeatMissing("typedDependencies", "edu.uchsc.ccp.uima.annotation.syntactic.CCPTokenAnnotation");
147        if (lowLevelTypeChecks)
148          ll_cas.ll_setRefArrayValue(ll_cas.ll_getRefValue(addr, casFeatCode_typedDependencies), i, v, true);
149        jcas.checkArrayBounds(ll_cas.ll_getRefValue(addr, casFeatCode_typedDependencies), i);
150        ll_cas.ll_setRefArrayValue(ll_cas.ll_getRefValue(addr, casFeatCode_typedDependencies), i, v);
151      }
152     
153    
154    
155    
156      /** initialize variables to correspond with Cas Type and Features
157            * @generated */
158      public CCPTokenAnnotation_Type(JCas jcas, Type casType) {
159        super(jcas, casType);
160        casImpl.getFSClassRegistry().addGeneratorForType((TypeImpl)this.casType, getFSGenerator());
161    
162     
163        casFeat_partOfSpeech = jcas.getRequiredFeatureDE(casType, "partOfSpeech", "uima.cas.String", featOkTst);
164        casFeatCode_partOfSpeech  = (null == casFeat_partOfSpeech) ? JCas.INVALID_FEATURE_CODE : ((FeatureImpl)casFeat_partOfSpeech).getCode();
165    
166     
167        casFeat_stem = jcas.getRequiredFeatureDE(casType, "stem", "uima.cas.String", featOkTst);
168        casFeatCode_stem  = (null == casFeat_stem) ? JCas.INVALID_FEATURE_CODE : ((FeatureImpl)casFeat_stem).getCode();
169    
170     
171        casFeat_lemma = jcas.getRequiredFeatureDE(casType, "lemma", "uima.cas.String", featOkTst);
172        casFeatCode_lemma  = (null == casFeat_lemma) ? JCas.INVALID_FEATURE_CODE : ((FeatureImpl)casFeat_lemma).getCode();
173    
174     
175        casFeat_tokenNumber = jcas.getRequiredFeatureDE(casType, "tokenNumber", "uima.cas.Integer", featOkTst);
176        casFeatCode_tokenNumber  = (null == casFeat_tokenNumber) ? JCas.INVALID_FEATURE_CODE : ((FeatureImpl)casFeat_tokenNumber).getCode();
177    
178     
179        casFeat_typedDependencies = jcas.getRequiredFeatureDE(casType, "typedDependencies", "uima.cas.FSArray", featOkTst);
180        casFeatCode_typedDependencies  = (null == casFeat_typedDependencies) ? JCas.INVALID_FEATURE_CODE : ((FeatureImpl)casFeat_typedDependencies).getCode();
181    
182      }
183    }
184    
185    
186    
187
```
